# Familial Status Modulates the Stability of Excellent Response in Non-Medullary Thyroid Carcinoma: Implications for Tailored Surveillance

**DOI:** 10.3390/cancers18101525

**Published:** 2026-05-09

**Authors:** Laura Valerio, Alfonso Sagnella, Fabio Maino, Elisa Mattii, Alessandra Cartocci, Maria Grazia Castagna

**Affiliations:** 1 Department of Medical, Surgical and Neurological Sciences, University of Siena, Viale Bracci 16, 53100 Siena, Italy; lau.val@hotmail.it (L.V.); alfonso.sagnella@student.unisi.it (A.S.); fabio.maino@ao-siena.toscana.it (F.M.); elisa.mattii1@gmail.com (E.M.); 2 Department of Molecular and Developmental Medicine, University of Siena, 53100 Siena, Italy; alessandra.cartocci@dbm.unisi.it

**Keywords:** differentiated thyroid carcinoma, ATA risk classes, familial non medullary thyroid carcinoma, thyroid cancer outcome, dynamic risk stratification

## Abstract

Background: The clinical impact of familial history on the prognosis of non-medullary thyroid cancer (NMTC) remains controversial. This study aimed to investigate whether familial NMTC (fNMTC) is associated with different patterns of response-to-therapy evolution over time compared to sporadic NMTC (sNMTC), using a dynamic risk stratification (DRS) approach. Methods: We analyzed 665 sNMTC cases and 130 fNMTC cases. Response to therapy was assessed at the first follow-up (6–12 months after initial therapy) and at the last outcome. Univariate and multivariate analyses were used to assess the role of family history as a modifier of response reclassification, independently of established prognostic factors. Results: A significant difference in the dynamic pattern of response evolution was observed between fNMTC and sNMTC (*p* = 0.003), with familial cases showing higher response variability. Among patients with an initial excellent response (n = 558), familial status remained the only independent predictor of losing excellent response (OR 3.3, 95% CI 1.54–7.12; *p* = 0.002). The worsening in fNMTC was primarily driven by transitions to indeterminate or biochemical incomplete responses (12.3% vs. 4.2%, *p* = 0.006), while structural progression remained rare and similar between groups (2.5% vs. 1.3%, *p* = 0.33). Regarding recovery, 50.2% of patients with an initial non-excellent response achieved an excellent response, with no significant difference between familial and sporadic cases (61.2% vs. 47.3%, *p* = 0.1). An intermediate-to-high ATA risk class was independently associated with a lower probability of achieving an excellent response (OR 0.45, 95% CI 0.25–0.91; *p* = 0.01). Conclusions: These findings suggest that “Excellent Response” is more fragile in a familial context. Familial NMTC might require prolonged and vigilant biochemical surveillance, even when cases appear to be in full remission.

## Simple Summary

This study evaluated the prognostic role of family history in non-medullary thyroid cancer (NMTC) by analyzing dynamic risk stratification (DRS) patterns in familial (fNMTC) versus sporadic (sNMTC) cases. In a cohort of 795 patients, fNMTC showed significantly greater variability in the response to therapy over time. Notably, among patients achieving an initial excellent response, familial status independently predicted a higher risk of response variability, mainly toward indeterminate or biochemical incomplete responses, while structural disease progression remained uncommon and comparable between groups. Conversely, the likelihood of achieving an excellent response from a non-excellent initial status was not significantly influenced by family history but was reduced in patients with intermediate-to-high ATA risk. Overall, these findings indicate that an excellent response is less stable in familial NMTC, supporting the need for prolonged and careful biochemical monitoring in this subgroup.

## 1. Introduction

Dynamic risk stratification (DRS) based on response-to-therapy assessments has represented a major advance in the management of patients with differentiated thyroid carcinoma (DTC). By integrating biochemical, structural, and imaging findings obtained during follow-up, DRS allows risk estimates to be continuously updated beyond initial staging systems. This approach provides a more accurate and individualized prediction of long-term outcomes, guiding the intensity of surveillance and treatment strategies [1,2].

Unlike traditional static risk models, which rely primarily on baseline clinicopathological features assessed at diagnosis, DRS reflects the biological behavior of the disease over time and identifies the heterogeneity of individual patient characteristics. In clinical practice, this means that patients initially classified as intermediate or high risk may later demonstrate an excellent response and require less intensive follow-up, whereas others initially considered low risk may show biochemical or structural evidence of disease persistence, warranting closer monitoring and additional therapeutic interventions. This dynamic reassessment has therefore become a cornerstone of personalized medicine in thyroid oncology, improving both prognostic precision and resource allocation.

More recently, several studies have investigated additional factors, including sex, the initial American Thyroid Association (ATA) risk category and serum thyroglobulin levels, which significantly influence changes in the initial response to therapy over time [3,4,5].

However, the role of the family history of thyroid cancer has never been specifically addressed within a dynamic risk stratification framework. Familial non-medullary thyroid carcinoma (fNMTC) accounts for a non-negligible proportion of DTC cases, yet its prognostic role remains incompletely defined.

Familial disease is generally defined by the occurrence of non-medullary thyroid carcinoma in two or more first-degree relatives, in the absence of other known hereditary syndromes associated with thyroid malignancy. Although fNMTC represents only a minority of all thyroid cancer cases, its identification has important implications not only for prognosis, but also for the clinical surveillance of affected families and for understanding the possible genetic and molecular mechanisms underlying tumor development. The absence of clearly established susceptibility genes in most non-syndromic familial cases has further contributed to the uncertainty surrounding its biological significance and clinical management.

Previous studies have primarily focused on the clinical and pathological presentation at diagnosis, reporting conflicting results [6,7]. While some authors have described a higher prevalence of multifocality, lymph node involvement, or more advanced disease at presentation in familial cases [8,9,10,11,12,13,14,15,16,17,18,19,20,21], others have failed to confirm a more aggressive phenotype when compared with sporadic NMTC [7,22,23,24].

Similarly, studies evaluating the risk of persistence or recurrence during follow-up in patients with fNMTC have yielded inconsistent findings, with reports ranging from increased recurrence rates to outcomes comparable to sporadic disease [9,11,13,25,26].

This inconsistency may be partly explained by differences in study design, variable definitions of familial disease, retrospective data collection, and heterogeneity in treatment strategies across cohorts. In addition, many studies have included relatively small sample sizes, limiting statistical power and making comparisons across studies particularly difficult. As a result, whether fNMTC should be considered a distinct clinical entity characterized by an intrinsically more aggressive behavior remains a matter of ongoing debate.

Importantly, all available evidence on fNMTC has been derived from static outcome analyses, largely focused on recurrence or persistence as binary endpoints, without considering the dynamic evolution of response-to-therapy categories over time.

To date, no study has investigated whether a family history of DTC independently influences the longitudinal trajectory of treatment response, when assessed through DRS and adjusted for established prognostic factors. The aim of this study was to investigate whether fNMTC, compared with sporadic disease, is associated with different patterns of response-to-therapy evolution over time. Specifically, we assessed the role of family history as a potential modifier of response reclassification, independently of established prognostic factors. Addressing this gap may provide novel insights into the biological and clinical behavior of fNMTC and help refine personalized follow-up strategies within a dynamic risk-based approach. A better understanding of this relationship could also support more tailored recommendations regarding the frequency of biochemical testing, imaging surveillance, and long-term management in patients with familial disease. Ultimately, clarifying whether family history carries independent prognostic significance within a DRS model may contribute to improving both risk communication and individualized patient care in differentiated thyroid carcinoma.

## 2. Materials and Methods

This was a retrospective study including 795 consecutive patients diagnosed with NMTC, followed at a tertiary referral center between 1990 and 2021. We excluded medullary, poorly differentiated and anaplastic thyroid cancer patients. Patients with incomplete clinical records, insufficient follow-up data, or unavailable information regarding the family history of thyroid cancer were also excluded from the analysis, in order to ensure reliable classification and longitudinal assessment of treatment response. The study population therefore consisted exclusively of patients with differentiated non-medullary thyroid carcinoma and adequate follow-up allowing for dynamic response evaluation over time.

All patients were longitudinally monitored with a follow-up schedule individualized according to disease status and risk stratification. Initial treatment consisted of thyroid surgery, followed or not by radioactive iodine (RAI) therapy according to individual risk assessment and clinical indications. The indication for postoperative RAI administration was based on tumor characteristics, extent of disease, histopathological findings, and the estimated risk of recurrence, in line with evolving international guidelines [27].

At each follow-up visit, patients underwent a comprehensive clinical evaluation including serum hormonal assessment of thyroglobulin (Tg), thyroid-stimulating hormone (TSH), and anti-thyroglobulin antibodies (AbTg), together with high-resolution neck ultrasonography. Additional imaging procedures, such as a diagnostic whole-body scan, computed tomography (CT), magnetic resonance imaging (MRI), positron emission tomography (PET), or fine-needle aspiration cytology were performed in cases of suspected or confirmed persistent or recurrent disease, in accordance with standard clinical practice and guideline recommendations [27].

### 2.1. Study Population

We retrospectively evaluated 665 sNMTC and 130 fNMTC patients followed at the Endocrine Unit of the Siena University Hospital, Italy. Patients were classified as having fNMTC when at least one first-degree relative had a confirmed diagnosis of non-medullary thyroid carcinoma, in the absence of known hereditary syndromes associated with thyroid malignancies. All remaining cases were classified as sNMTC.

Response to therapy was assessed at the first follow-up (6–12 months after initial therapy) and at the last follow-up. For each patient, longitudinal changes in response categories were recorded in order to evaluate possible reclassifications over time, including the improvement, stability, or worsening of the initial response.

The study was approved by the local Ethics Committee (Comitato Etico Area Vasta Sud-Est) (Prot. N. 10167). The study was conducted in accordance with the provisions of the Declaration of Helsinki (2013). All patients provided written informed consent for the use of their data for scientific purposes.

### 2.2. Definition of Familial NMTC

Familial NMTC was defined by the occurrence of differentiated thyroid carcinoma in two (81% of our cases) or more first-degree relatives (19% of our cases), in the absence of other hereditary cancer syndromes known to involve the thyroid gland.

### 2.3. Criteria Used to Define the Clinical Status of NMTC Patients

Response to initial treatment was defined according to the 2015 ATA criteria [27], classifying patients into Excellent Response (ER): no clinical, biochemical or structural evidence of disease; Indeterminate Response (IR): nonspecific biochemical or structural findings, which cannot be confidently classified as either benign or malignant (this includes patients with stable or declining AbTg levels without definitive structural evidence of disease); Biochemical Incomplete Response (BIR): abnormal Tg or rising AbTg levels in the absence of localizable disease; Structural Incomplete Response (SIR): persistent or newly identified locoregional or distant metastases on imaging. The same response-to-therapy classification was systematically applied at the last follow-up evaluation, allowing for a dynamic reassessment of response status over time.

### 2.4. Statistical Analysis

Continuous variables are reported as mean ± standard deviation or median and interquartile range, according to the normality of the distribution tested by Kolmogorov–Smirnov test, and were compared using the Student’s *t* test or the Mann–Whitney U test. Categorical variables are expressed as absolute numbers and percentages and were compared using the χ^2^ test or Fisher’s exact test, as appropriate.

Changes in response categories over time were evaluated as the maintenance of or transition to a different response category. Odds ratios (ORs) with 95% confidence intervals (CIs) were calculated to assess the association between familial status and response category changes.

Univariate and multivariate logistic regression models were used to identify factors associated with the loss of an excellent response among patients with an excellent response at first follow-up and recovery to excellent response among patients with a non-excellent initial response (indeterminate response, biochemical incomplete response and structural incomplete response). Statistical analysis was performed using the SPSS Statistics version 22.0. A *p*-value < 0.05 was considered statistically significant.

## 3. Results

### 3.1. Study Population

The study cohort included 795 patients with NMTC, with a median age at diagnosis of 45 years (interquartile range [IQR] 34–59), and a predominance of female patients (73%). Most tumors were diagnosed at an early local stage (64.7% of patients had T1–T2 disease) while lymph node metastases were present in 24.8% of NMTC cases. Extrathyroidal extension, multifocality, and bilaterality were observed in a substantial proportion of patients, reflecting the heterogeneous pathological presentation of the disease. The fNMTC group represented 16.4% (130/795) of the overall cohort. Most patients underwent a total thyroidectomy (73.9%), and radioiodine therapy was administered in 83.1% of cases. At the first follow-up evaluation, an excellent response to therapy was achieved in approximately 70% of patients. During the long-term follow-up, the proportion of patients with an excellent response increased to 80%. The median follow-up duration was 12.6 years (Table 1).

### 3.2. Dynamic Evolution of Response to Therapy

**Figure 1:**
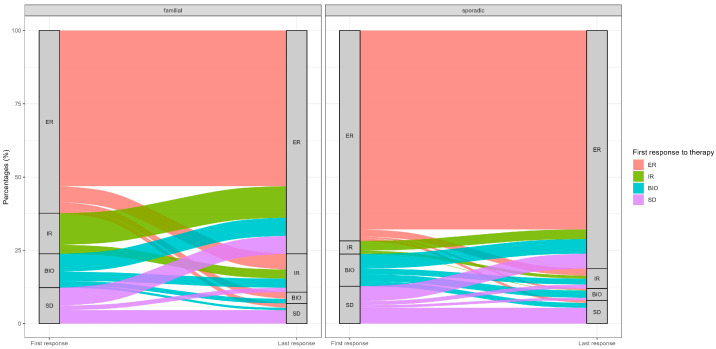
Dynamic pattern of response evolution in *f*NMTC and *s*NMTC.

When the entire cohort was considered (n = 795), response evolution from the first to the last follow-up showed a significant difference in the dynamic pattern of response between familial and sporadic NMTC (*p* = 0.003). The Alluvial plot highlights a more dynamic pattern of response evolution in fNMTC, characterized by both favorable and unfavorable transitions over time, whereas sNMTC more commonly exhibited response stability between the first and last follow-up assessments (Figure 1).

### 3.3. Loss of Excellent Response over Time

Among patients with an excellent response at the first follow-up (n = 558), the loss of an excellent response at the final outcome occurred significantly more frequently in familial than in sporadic NMTC (*p* < 0.001) (Table 2). To further explore the clinical significance of these transitions, response categories were dichotomized into the maintenance of an excellent response versus any shift to a non-excellent response (indeterminate, biochemical incomplete, or structural incomplete). Patients with familial disease were more likely to lose their excellent response over time, after a median follow up of 5 years, compared with sporadic cases (after a median follow up of 9 years) (14.8% vs. 5.5%), corresponding to a three-fold higher risk of non-excellent outcomes (OR 3.02, 95% CI 1.45–6.26; *p* = 0.004). Nevertheless, when the loss of an excellent response was decomposed according to the type of worsening, familial cases showed a higher rate of biochemical incomplete response compared with sporadic cases (3.7% vs. 0.8%; OR 4.55, 95% CI 1.0–20.3; *p* = 0.03). Similarly, the transition to an indeterminate response was more frequent in familial disease (8.6% vs. 3.4%; OR 2.7, 95% CI 1.1–6.6; *p* = 0.02). In contrast, progression to structural disease was rare and did not significantly differ between the two groups (2.5% in fNMTC and 1.3% in sNMTC, *p* = 0.33).

At univariate analysis the loss of an excellent response was associated with familial disease (OR 3.02, 95% CI 1.45–6.26, *p* = 0.004), extrathyroidal invasion (OR 3.06, 95% CI 1.57–5.96, *p* = 0.001), multifocality (OR 3.18, 95% CI 1.62–6.26, *p* = 0.001), bilaterality (OR 3.36, 95% CI 1.72–6.56, *p* < 0.001), the ATA risk class (OR for intermediate/high risk patients 3.0, 95% CI 1.52–5.94, *p* = 0.002) and initial radioiodine treatment (OR 3.28, 95% CI 0.99–10.85, *p* = 0.039). Familial disease remained the only independent predictor of the loss of an excellent response during follow-up (OR 3.31, 95% CI 1.54–7.12; *p* = 0.002) (Table 3).

### 3.4. Evolution from Non-Excellent Response to Excellent Response According to Familial Status

Among patients with a non-excellent response at first evaluation, 119/237 (50.2%) subsequently achieved an excellent response during follow-up, without differences between familial and sporadic NMTC (61.2% of familial and 47.3% of sporadic NMTC cases, *p* = 0.11).

In the subgroup of patients with an indeterminate response at the first follow-up, the probability of transitioning to an excellent response was similar in familial and sporadic disease (77.8% vs. 73.3%, *p* = 0.75). Likewise, among patients starting with a biochemical incomplete response, transitions toward an excellent response occurred in 53.3% of familial and 45.2% of sporadic cases (*p* = 0.58). In patients with structural disease at the first follow-up, improvement to an excellent response occurred in 50.0% of familial and 40.0% of sporadic cases, while persistence of structural disease was observed in 37.5% and 42.4%, respectively, without significant differences (*p* = 0.58) (Table 4). In univariate analysis, only the ATA low-risk class was significantly associated with a higher likelihood of response improvement (OR 0.45 for high/intermediate vs. low risk, 95% CI 0.25–0.91; *p* = 0.011). Familial disease showed a non-significant trend toward greater improvement compared with sporadic cases (OR 1.76, *p* = 0.116) (Table 5).

## 4. Discussion

The results of this study provide a novel perspective on the clinical behavior of NMTC and suggest that familial status is associated with differences in response-to-therapy evolution over time. The main finding of this study suggests that a familial history in NMTC acts as an independent predictor of the loss of ER over time. Our longitudinal analysis suggests a tendency for fNMTC to show a more ‘volatile’ clinical course, with a higher probability of reclassification toward less favorable response categories. Patients with fNMTC who achieve an ER at the first follow-up are over three times more likely to lose this status compared to their sporadic counterparts (OR 3.56, 95% IC 1.70–7.48). Interestingly, this association remained significant even after adjusting for features such as age, gender, ATA risk class, multifocality, bilaterality, extrathyroidal extension and radioiodine treatment. These findings suggest that familial status is associated with biological factors predisposing to the late loss of an excellent response, which may not be entirely captured by current clinicopathological staging systems. In the era of DRS, an ER is typically considered a hallmark of “remission” with a very low risk of recurrence (1–4%) [1,2,28,29]. Conversely, our results suggest that in fNMTC, the ER status is more fragile. Nevertheless, when we decomposed the “loss of ER”, we found that the worsening in fNMTC was primarily driven by transitions to indeterminate or biochemical incomplete responses (12.3% vs. 4.2%, *p* = 0.006), while structural progression remained rare and similar between groups (2.5% vs. 1.3%, *p* = 0.33). Previous matched-cohort studies reported that familial non-medullary thyroid carcinoma does not confer a worse structural recurrence risk [26,30]. Consistently, we did not observe a clear increase in overt structural disease. Nevertheless, our findings complement previous recurrence-based studies [26,30] by showing that response dynamics may be more sensitive than structural recurrence in detecting subtle differences in disease behavior between familial and sporadic cases.

From a clinical perspective, our findings are reassuring, indicating that although patients with fNMTC more frequently exhibit fluctuating or rising Tg levels or nonspecific ultrasound findings, they do not necessarily carry a higher risk of developing structural disease.

Several studies have begun to explore the instability of an ER over time [1,2,29,31]. Investigations from the Memorial Sloan Kettering group have shown that approximately 2–5% of patients with differentiated thyroid carcinoma lose their ER status during follow-up [1]. In our cohort, we observed a markedly higher rate of ER loss among familial cases (14.8%), whereas the rate observed in sporadic NMTC was consistent with previously reported data (5.5%). These findings suggest that a familial history may act as a “silent” risk factor that is not fully captured by current dynamic risk stratification models, particularly in the early phases of follow-up.

A crucial finding of our study is that the “disadvantage” of fNMTC appears to be limited to the stability of remission, rather than the response to cancer therapies. We observed that the probability of transitioning from a non-excellent to an excellent response was comparable between familial and sporadic cases (61.2% vs. 47.3%, *p* = 0.1). In this context, the initial ATA risk category remained the only significant predictor of recovery (OR 0.45, 95% IC 0.25–0.91). This indicates that once the disease is persistent, the possibility of it being “cured” or controlled by therapy depends more on the biological characteristics of the tumor (stage, invasiveness) than on the familial background.

Accordingly, these findings do not appear to justify more aggressive initial surgery or routine radioactive iodine therapy in all familial cases. Rather, they suggest the potential value of an approach based on ‘enhanced vigilance’ rather than ‘enhanced intervention. The clinical focus should shift toward a more tailored and prolonged follow-up strategy, ensuring that these low-grade biochemical shifts are detected early. Since the “treatability” of the disease remains high, a stricter monitoring approach allows for timely management only when necessary, avoiding the risks of overtreatment while maintaining optimal long-term oncological safety. Ultimately, recognizing the specific evolutionary pattern of fNMTC may support a more personalized ‘precision medicine’ approach, ensuring that the apparent safety of an initial remission does not preclude careful long-term follow up.

This study possesses several key strengths that reinforce the validity of our findings. Most notably, it is one of the first to utilize a longitudinal DRS approach to compare familial and sporadic NMTC, allowing us to capture the unique temporal instability of the familial form rather than relying on static outcomes. Furthermore, differentiating biochemical from structural progression allows for a more detailed assessment of disease evolution and raises the possibility that familial status may have independent prognostic relevance. However, certain limitations must be acknowledged. The retrospective design may introduce inherent selection or reporting biases. Additionally, as the study was conducted at a tertiary referral center, the cohort may reflect a more closely monitored population, potentially influencing the frequency of response reclassifications compared to general clinical settings. Moreover, the majority of fNMTC cases were represented by the occurrence of differentiated thyroid carcinoma in only two first-degree relatives.

## 5. Conclusions

In conclusion, our findings suggest that a familial history may influence the clinical course of NMTC, indicating that a uniform interpretation of an ER might not adequately reflect disease evolution in all cases. Patients with fNMTC who achieve an ER may require more prolonged surveillance due to their higher likelihood of transitioning to indeterminate or biochemical incomplete responses. Although fNMTC does not appear to increase the risk of structural relapses, its dynamic instability supports the need for a personalized, long-term follow-up strategy.

## Figures and Tables

**Table 1:** Clinical-pathological characteristics and response to therapy of the study population.

Characteristic	Study Population (n = 795)
Age at diagnosis, years	
Median (IQR)	45 (37–59)
Male sex	215 (27.0%)
T stage	
T1–T2	515 (64.7%)
T3–T4	280 (35.3%)
N stage	
N1 disease	197 (24.8%)
Extrathyroidal invasion	255 (32.0%)
Multifocality	297 (37.3%)
Bilaterality	211 (26.5%)
Familial NMTC	130 (16.4%)
Total thyroidectomy	588 (73.9%)
Radioiodine (RAI) therapy	661 (83.1%)
Response to therapy at first follow-up	
Excellent response	558 (70.2%)
Non-excellent response	237 (29.8%)
Response to therapy at last follow-up	
Excellent response	639 (80.3%)
Non-excellent response	156 (19.7%)
Follow-up, years	
Median (IQR)	12.6 (8.4–18.1)

**Table 2:** Evolution of DTC patients with excellent response at first follow-up according to familial or sporadic disease.

Last Response to Therapy	Familial (n = 81)	Sporadic (n = 477)	*p*-Value
Excellent response	69 (85.2%)	451 (94.5%)	
Indeterminate response	7 (8.6%)	16 (3.4%)	
Biochemical incomplete response	3 (3.7%)	4 (0.8%)	
Structural incomplete response	2 (2.5%)	6 (1.3%)	
Overall comparison			<0.001

**Table 3:** Factors associated with loss of excellent response at last follow-up: univariate and multivariable logistic regression analyses.

Variable	Category	Univariate OR (95% CI)	*p*-Value	Adjusted OR (95% CI)	*p*-Value
Disease type	Familial vs. Sporadic	3.02 (1.45–6.26)	0.004	3.31 (1.54–7.13)	0.002
Extrathyroidal invasion	Yes vs. No	3.06 (1.57–5.96)	0.001	1.56 (0.54–4.47)	0.412
Multifocality	Yes vs. No	3.18 (1.62–6.26)	0.001	2.06 (0.76–5.54)	0.153
Bilaterality	Yes vs. No	3.36 (1.72–6.56)	<0.001	1.58 (0.58–4.30)	0.367
ATA risk class	High/Intermediate vs. Low	3.00 (1.52–5.94)	0.002	1.94 (0.66–5.68)	0.226
Age at diagnosis (years)	Continuous	1.00 (0.98–1.02)	0.957	—	—
Histology	Follicular vs. Papillary	0.36 (0.04–3.15)	0.882	—	—
Gender	Male vs. Female	1.52 (0.75–3.11)	0.335	—	—
Radioiodine treatment	Yes vs. No	3.28 (0.99–10.85)	0.039	2.3 (0.61–8.66)	0.218

**Table 4:** Transition of non-excellent responses between first and last follow-up according to familial or sporadic disease.

			Last Follow-Up			
First Follow-Up	Group	Excellent Response	Indeterminate Response	Biochemical Incomplete Response	Structural Incomplete Response	Total
Biochemical incomplete response (BIR)	Familial	8 (53.3%)	4 (26.7%)	2 (13.3%)	1 (6.7%)	15
	Sporadic	33 (45.2%)	13 (17.8%)	16 (21.9%)	11 (15.1%)	73
Indeterminate response (IR)	Familial	14 (77.8%)	4 (22.2%)	0 (0.0%)	0(0.0%)	18
	Sporadic	22 (73.3%)	7 (23.3%)	1 (3.3%)	0(0.0%)	30
Structural incomplete response (SIR)	Familial	8 (50.0%)	2 (12.5%)	0(0.0%)	6 (37.5%)	16
	Sporadic	34 (40.0%)	9 (10.6%)	6 (7.1%)	36 (42.4%)	85

**Table 5:** Factors predicting achievement of excellent response at last follow-up among patients with non-excellent response at first follow-up.

Variable	Comparison	OR (95% CI)	*p*-Value
Disease type	Familial vs. Sporadic	1.76 (0.92–3.34)	0.116
Age at diagnosis (years)	Continuous	0.99 (0.97–1.00)	0.131
Extrathyroidal extension	Yes vs. No	0.65 (0.39–1.09)	0.135
Multifocality	Yes vs. No	0.89 (0.53–1.48)	0.749
Bilaterality	Yes vs. No	0.76 (0.45–1.30)	0.387
Histology	Follicular vs. Papillary	3.05 (0.12–75.7)	0.997
ATA risk class	High/Intermediate vs. Low	0.45 (0.25–0.91)	0.011
Gender	Female vs. Male	0.58 (0.34–1.00)	0.068

## Data Availability

The data presented in this study are available on request from the corresponding author. The data are not publicly available due to patients’ privacy.
